# Leaf Phosphorus Concentration Regulates the Development of Cluster Roots and Exudation of Carboxylates in *Macadamia integrifolia*

**DOI:** 10.3389/fpls.2020.610591

**Published:** 2021-01-13

**Authors:** Xin Zhao, Yang Lyu, Kemo Jin, Hans Lambers, Jianbo Shen

**Affiliations:** ^1^Department of Plant Nutrition, College of Resources and Environmental Sciences, Key Laboratory of Plant-Soil Interactions, Ministry of Education, National Academy of Agriculture Green Development, China Agricultural University, Beijing, China; ^2^The UWA Institute of Agriculture, The University of Western Australia, Perth, WA, Australia; ^3^School of Biological Sciences, The University of Western Australia, Perth, WA, Australia

**Keywords:** cluster roots, critical phosphorus concentration, exudation, Proteaceae, phosphorus supply

## Abstract

Phosphorus (P) deficiency induces cluster-root formation and carboxylate exudation in most Proteaceae. However, how external P supply regulates these root traits in *Macadamia integrifolia* remains unclear. Macadamia plants were grown hydroponically with seven P levels to characterize biomass allocation, cluster-root development, and exudation of carboxylates and acid phosphatases. Plant biomass increased with increasing P supply, peaking at 5 μM P, was the same at 5–25 μM P, and declined at 50–100 μM P. Leaf P concentration increased with increasing P supply, but shoot biomass was positively correlated with leaf P concentration up to 0.7–0.8 mg P g^–1^ dry weight (DW), and declined with further increasing leaf P concentration. The number of cluster roots declined with increasing P supply, with a critical value of leaf P concentration at 0.7–0.8 mg P g^–1^ DW. We found a similar trend for carboxylate release, with a critical value of leaf P concentration at 0.5 mg g^–1^ DW, but the activity of acid phosphatases showed a gradually-decreasing trend with increasing P supply. Our results suggest that leaf P concentration regulates the development and functioning of cluster roots, with a critical P concentration of 0.5–0.8 mg g^–1^, above which macadamia growth is inhibited.

## Introduction

Phosphorus (P) is an essential nutrient for all life on the earth (Baker et al., 2015; Cong et al., 2020). Phosphorus in soil has a very low solubility and mobility. In acid soils, most P is sorbed by Al- or Fe-oxides and -hydroxides (Parfitt, 1989; Shen et al., 2011), while it is precipitated with calcium (Larsen, 1967) and sorbed onto clay minerals (Devau et al., 2010) in neutral-to-calcareous soils. The availability of P in soil limits plant growth in many agricultural systems (Marschner, 1995; Cong et al., 2020). Thus, plant strategies to efficiently acquire P from soil play important roles in increasing plant growth and yield (Shen et al., 2011). One of the most important strategies for increasing P acquisition includes root morphological modifications, involving higher root/shoot ratio, more root branching, greater root hair length, faster root elongation, and an increased ratio of fine roots (Shane et al., 2003a; Lynch, 2015; Wen et al., 2017).

Most Proteaceae grow on nutrient-impoverished soils with low P availability, especially in Australia and South Africa (Lamont, 2003; Lambers et al., 2010, 2015b) and evolved specialized root structures—cluster roots (Lambers et al., 2015a). Cluster roots, originally called “proteoid roots,” were first noted by Engler (1894) and described in detail by Purnell (1960) as “dense clusters of rootlets of limited growth” along lateral roots. The specialized morphology of cluster roots enhances root surface area (Shen et al., 2011; Lambers et al., 2015a), and most importantly, cluster roots release substantial amounts of carboxylates (Gardner et al., 1982; Dinkelaker et al., 1989; Keerthisinghe et al., 1998; Watt and Evans, 1999; Lambers et al., 2002), mostly citrate and malate (Neumann and Martinoia, 2002; Shen et al., 2011), and thus mobilize soil P sources (Hoffland et al., 1989; Lambers et al., 2015a; Krishnapriya and Pandey, 2016). The release of carboxylates coincides with rhizosphere acidification, particularly in *Lupinus albus* (Gardner et al., 1982; Marschner et al., 1987; Neumann and Martinoia, 2002; Meyer et al., 2010). Acid phosphatases are also exuded into the rhizosphere, and these hydrolyze organic P (Gilbert et al., 1999; Wasaki et al., 2003; Delgado et al., 2015). Cluster roots are ephemeral structures (Lambers et al., 2015a), and live for about 20 days, from rootlet emergence to senescence in *Hakea prostrata* (Shane et al., 2004c). In white lupin, visible rootlets begin to develop on the apical regions of the lateral roots in the juvenile stage of cluster roots, followed by an exudative burst of large amounts of citrate and malate at the mature stage (Watt and Evans, 1999). Also, exudation of protons reaches a maximum at the mature stage of cluster roots, leading to an acidification of the rhizosphere (Zhu et al., 2005).

Macadamia (*Macadamia integrifolia*), which belongs to Proteaceae, is native to Australia (Hue, 2009), and is well adapted to low-P environments (Aitken et al., 1992). In macadamia, previous studies reported some descriptive relationships between the leaf P/soil P and dry weight (DW) of macadamia seedlings (Aitken et al., 1992, 1993; Stephenson et al., 2002). Cluster root growth (as a percentage of total root weight) of macadamia is related to the soil P concentration and inhibited at high soil P levels (Aitken et al., 1992). Moreover, excessive P application suppresses the growth of macadamia trees (Aitken et al., 1993), and causes leaf chlorosis (Hue and Nakamura, 1988; Nagao et al., 1992; Gallagher et al., 2003). Hue (2009) evaluated the effects of P and Fe fertilizers and their interactions on the development of cluster roots in macadamia. Several authors showed that excess P supply causes P-toxicity symptoms in Proteaceae (Groves and Keraitis, 1976; Grose, 1989) such as *Banksia ericifolia* (Handreck, 1991; Parks et al., 2000), and *H. prostrata* (Shane et al., 2004a).

The formation of cluster roots and carboxylate exudation induced by low P availability have been studied extensively in *L. albus* (Shane et al., 2003b; Wasaki et al., 2003; Shen et al., 2005; Li et al., 2008; Cheng et al., 2014; Gallardo et al., 2019), and many species of Proteaceae like *Hakea* sp. (Lamont, 1972a,b), *Grevillea robusta* (Skene et al., 1996, 1998), *H. prostrata* (Shane et al., 2003a), *Grevillea crithmifolia* (Shane and Lambers, 2006), and *Euplassa cantareirae* (de Britto Costa et al., 2016). Yet, how variation in P supply affects these root traits, especially the functioning of cluster roots of macadamia, as a valuable nut tree, is poorly understood.

In this study, we tested the hypothesis that external P supply affects the internal shoot P concentration, and thus regulates cluster-root formation and carboxylate exudation in macadamia at a critical P value. This study aimed to provide valuable insights into the mechanism underlying cluster-root development and functioning for efficient P acquisition and thus underpin best nutrient management to increase P use efficiency and avoid P toxicity in macadamia cultivation.

## Materials and Methods

### Experimental Setup

The experiment was conducted in a greenhouse at China Agricultural University, Beijing (40° 1′ 46″ N, 116° 17′ 11″ E). Seeds of macadamia were collected in Yunnan Province, Southern China. Seed P concentration was 1.9 ± 0.6 mg P g^–1^ DW. Seeds were planted in washed sand and watered with deionized water for germination. Seedlings with four leaves were transplanted into half-strength nutrient solution which was modified to contain only 2.5 μM P for 2 weeks; after this, plants were transferred to 100% strength hydroponics and cotyledons were removed. Nutrient solutions were renewed every 7 days. There were seven P application rates: 0, 2.5, 5, 10, 25, 50, 100 μM supplied as KH_2_PO_4_; the K concentration was the same in every treatment, because KH_2_PO_4_ was replaced by KCl. All other basal nutrients were provided as follows: MgSO_4_ (500 μM); Ca(NO_3_)_2_ (2000 μM); K_2_SO_4_ (700 μM); Fe-EDTA (20 μM); H_3_BO_3_ (10 μM); MnSO_4_ (0.5 μM); ZnSO_4_ (0.5 μM); CuSO_4_ (0.2 μM); (NH_4_)_6_Mo_7_O_24_ (0.01 μM). The pot size was 7.8 L, and the initial pH of the nutrient solution was measured with a pH-sensitive microelectrode and adjusted to 5.8 using NaOH or HCl. There were four replicates in each treatment.

### Plant Harvest and Root Sampling

Plants were harvested after 6 months of growth. Plants were divided into roots, stems, young leaves, and old leaves for harvesting. The number of cluster roots was measured in every treatment. All plant samples were oven-dried at 70°C for 72 h to determine DW. Dried material was ground to a powder with a stainless-steel grinder to determine P concentration. Plant powders were digested using the microwave-accelerated reaction system (CEM, Matthews, NC, United States). We used 6 mL HNO_3_ and 2 mL 30% (v/v) H_2_O_2_ during the digestion process.

### Collection of Root Exudates and Measurement of Carboxylates

We used 0.5 g excised root segments in every treatment to collect root exudates before plants were harvested. We sampled the active white root cluster as cluster root segment. For the treatments without cluster roots, we sampled root tips bearing no cluster roots. Roots were washed with deionized water four times to remove ions from the root surface and xylem exudate. Then roots were incubated in a centrifuge tube with 3 mL incubation medium for 2 h to collect exudates. The composition of the incubation medium was (μM): MgCl_2_ (200), KCl (100), CaCl_2_ (600), and H_3_BO_3_ (5) and it was adjusted to the same pH as that of the nutrient solution with NaOH or HCl (5.8). After the collection of exudates, two drops of microbial inhibitor Micropur (Sicheres Trinkwasser, Munich, Germany) at 0.01 g L^–1^ and two drops of concentrated H_3_PO_4_ were added to inhibit microbial degradation of root exudates (Cheng et al., 2014; Wen et al., 2019).

Root exudates were stored at −20°C until the analysis of carboxylates by HPLC. Before analysis, root exudation samples were passed through sterile Millex GS Millipore 0.22-μm filters. The analysis method was the same as described by Shen et al. (2003) and Wang et al. (2007).

Cluster roots at different developmental stages were differentiated as shown in Section “Results” and taken from the plants grown in the 0 μM P treatment.

### Determination of Root-Released Acid Phosphatase Activity (APase)

Before plants were harvested, another 0.5 g root sample was taken to measure acid phosphatase (APase). Root samples were washed in deionized water four times. The APase activity on the root surface was analyzed according to Neumann et al. (1999). Root samples were placed in a centrifuge tube with 0.4 mL substrate buffer (pH 5.2); then 0.1 mL *p*-nitrophenyl phosphate (NPP) was added and 0.5 mL deionized water. Centrifuge tubes were placed in a 30°C water bath for 15 min, and then 0.5 mL 0.5 M NaOH was added to terminate the reaction and develop the color. The absorbance of the resulting color was determined spectrophotometrically (UV-2201, Shimadzu, Kyoto, Japan) at 405 nm.

### Determination of Nutrient Solution pH and Rhizosphere pH

In this experiment, we changed the nutrient solution every 7 days, and the solution pH was monitored every day using a pH-sensitive microelectrode (pH-HJ90B, Shanghai, China). We chose cluster-root segments to measure the rhizosphere pH of different development stages using an agar method with pH indicator. 0.75% w/v agar and 0.006% (w/v) bromocresol purple were mixed, and the pH was adjusted to 6.0. The agar was heated to boiling temperature and then cooled to 40°C.

Cluster roots were washed and placed in a clean Petri dish, and then liquid agar was poured into the Petri dish. After 10 min, the color along the roots changed, yellow representing acidification and purple alkalization, respectively.

### Data Analysis

One-way ANOVA was performed, and when appropriate, the *post hoc* means comparisons were done by SAS statistical software (8.1; SAS Institute, Inc., Cary, NC, United States). Data were analyzed by least squares fitting method and determined as non-linear regression functions in SigmaPlot 10.0 (United States). *P* < 0.05 was considered significant.

## Results

### Plant Growth and Biomass Allocation

**FIGURE 1:**
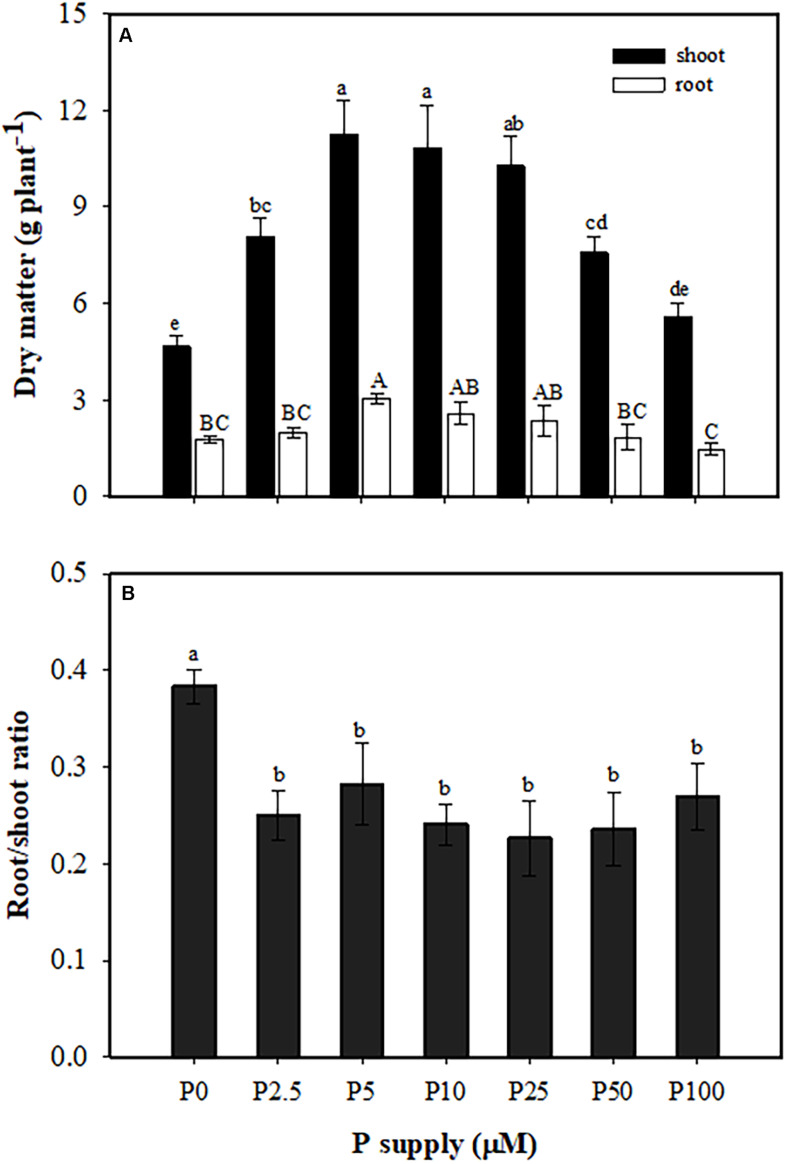
Partitioning of dry matter **(A)** and root/shoot ratio **(B)** of *Macadamia integrifolia* grown with different rates of phosphorus (P) supply. Plants were grown for 6 months at 0, 2.5, 5, 10, 25, 50, and 100 μM P (mean ± SE, *n* = 4). Different lowercase or uppercase letters denote a significant difference among plants grown with a different P supply (*P* < 0.05).

Plants produced more shoot biomass than root biomass in all treatments. Both shoot and root biomass increased with increasing P supply, with no further change from 5 to 25 μM P, and then growth was significantly inhibited at 50 and 100 μM P (Figure 1A). The greatest root/shoot ratio was found at 0 μM P, with a one-third decrease in all P treatments compared with no P supply (Figure 1B).

### Plant P Concentration and Content

**FIGURE 2:**
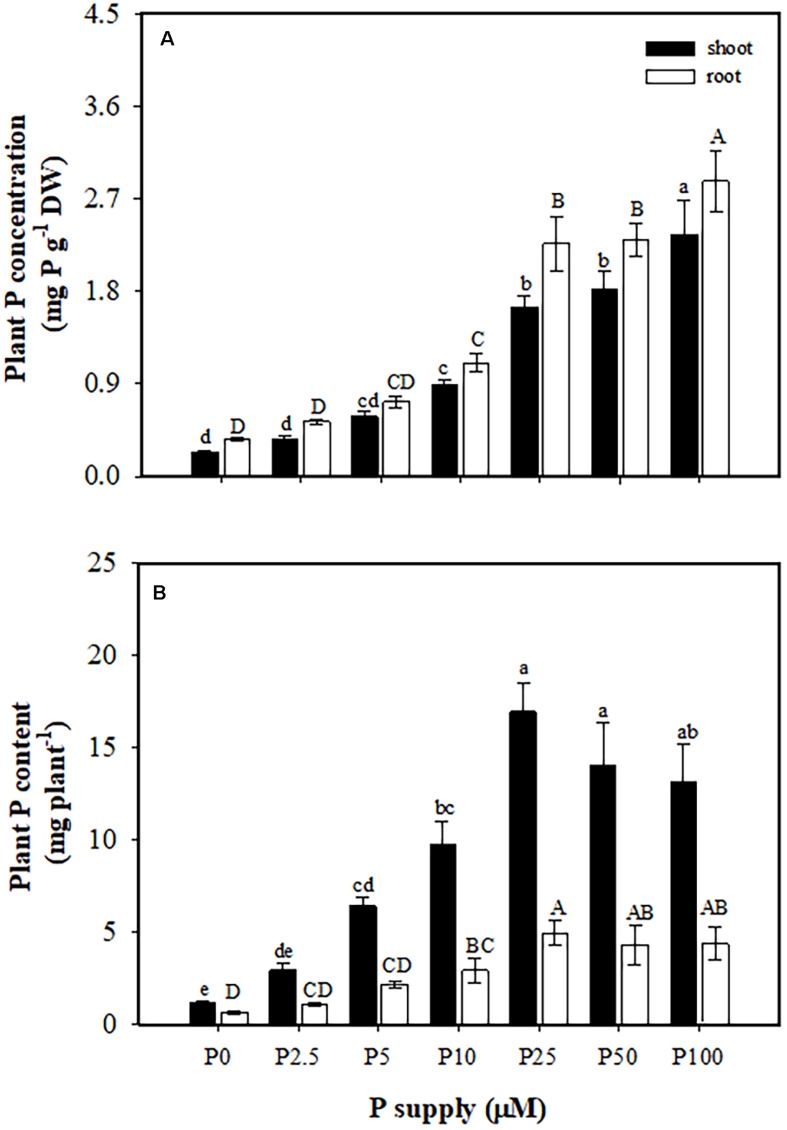
Phosphorus (P) concentration **(A)** and content **(B)** in shoots and roots of *Macadamia integrifolia*. Plants were grown for 6 months at 0, 2.5, 5, 10, 25, 50, and 100 μM P (mean ± SE, *n* = 4). Different lowercase or uppercase letters denote a significant difference among P supplies (*P* < 0.05).

Phosphorus concentrations in shoots (from 0.24 to 2.3 mg P g^–1^ DW) and roots (from 0.36 to 2.9 mg P g^–1^ DW) increased with increasing P supply (Figure 2A). The P content in shoots and roots also increased with increasing P supply from 0 to 25 μM, but showed no further increase at 25, 50, and 100 μM P (Figure 2B).

### Relationships Between External P Supply, Leaf P Concentration, and Shoot Biomass

**FIGURE 3:**
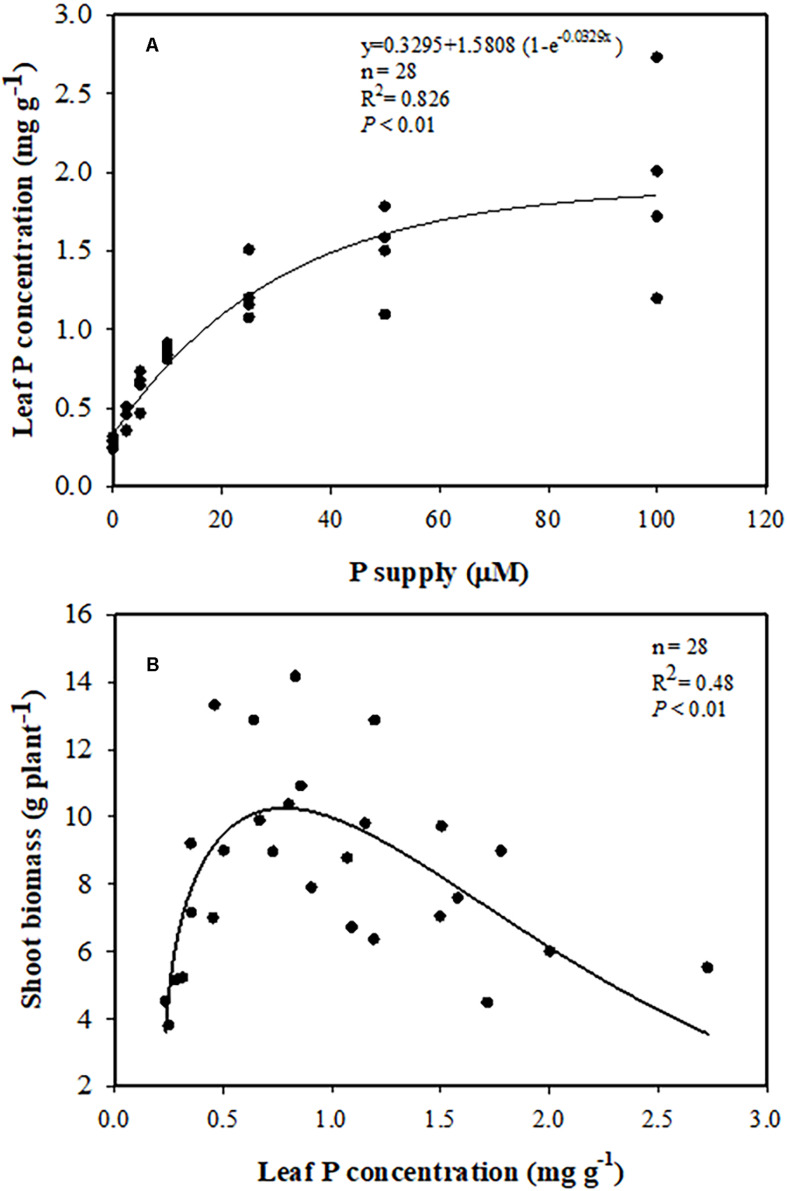
**(A)** Relationships between leaf phosphorus (P) concentration and P supply. **(B)** Relationship between shoot biomass and leaf P concentration.

Leaf P concentration had a positive correlation with external P supply. Leaf P concentration increased with increasing P supply, and reached 1.9 mg P g^–1^ DW, three times greater at 100 μM P than at 0 μM P (Figure 3A). Increasing P concentration in the leaves was associated with greater shoot biomass, which increased to a peak of 11 g DW plant^–1^ at a leaf P concentration of 0.7–0.8 P mg g^–1^ DW. However, with a further increase in leaf P concentration, shoot biomass gradually declined (Figure 3B). The same trend was found between shoot biomass and shoot P concentration (Supplementary Figure S1).

### Cluster-Root Formation and Exudation

**FIGURE 4:**
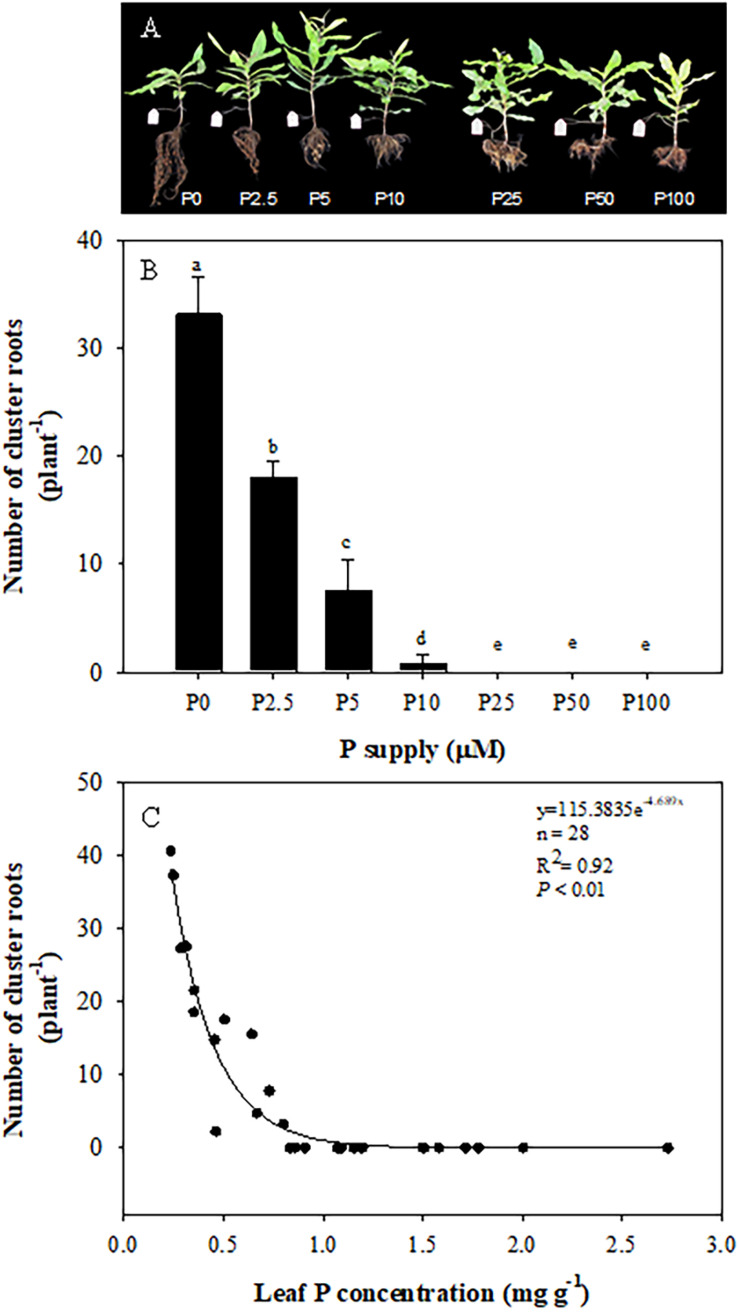
Effects of phosphorus (P) supply on the growth of *Macadamia integrifolia* **(A)** and number of cluster roots **(B)**, and the relationship between cluster root number and leaf P concentration **(C)**. The data in **(B)** represent the means of four replicates ± SE. Different letters indicate significant differences (*P* < 0.05).

As shown in Figure 4A, plants developed most cluster roots at 0 μM P, when roots were longer compared with + P treatments. There was a downward trend in the number of cluster roots with increasing P supply from 0 to 10 μM. Plants produced 33 cluster roots per plant on average at 0 μM P, but only 18 per plant at 2.5 μM P. At 5 μM P, the number decreased to eight per plant, and plants did not develop cluster roots when the P supply exceeded 10 μM P (Figure 4B). There was a significant negative correlation between the cluster-root number and leaf P concentration (Figure 4C).

**FIGURE 5:**
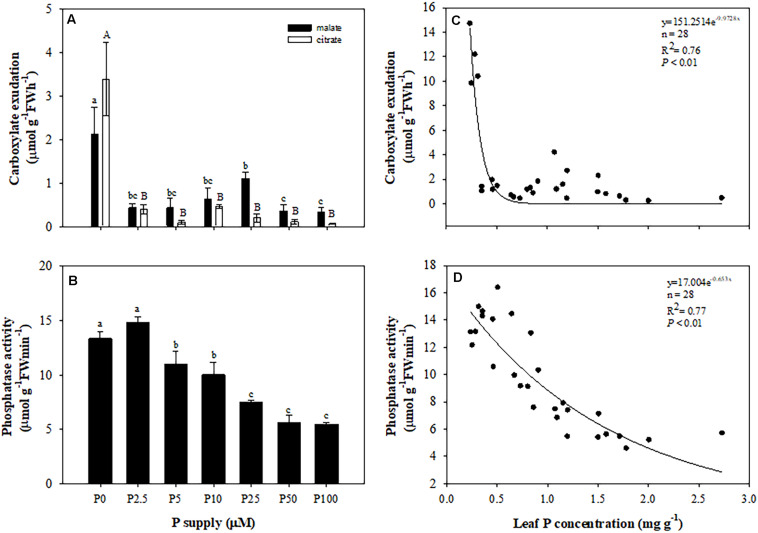
Effects of phosphorus (P) supply on root carboxylate exudation **(A)**, and the activity of acid phosphatase **(B)** of *Macadamia integrifolia*. Relationship between total carboxylates **(C)** and activity of acid phosphatase **(D)** and leaf P concentration. Plants were grown for 6 months at 0, 2.5, 5, 10, 25, 50, and 100 μM P. The data represent the means of four replicates ± SE. Different lowercase or uppercase letters indicate significant differences (*P* < 0.05).

**FIGURE 6:**
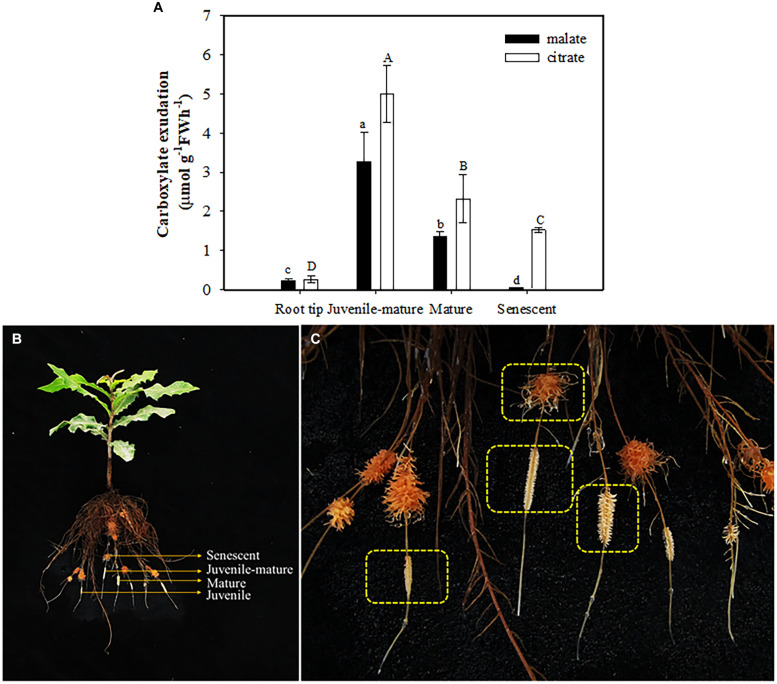
Rates of malate and citrate exudation **(A)** at different developmental stages **(B,C)** of cluster roots at 0 μM P supply. The data in the figure represent the means of four replicates ± SE. Different lowercase or uppercase letters indicate significant differences (*P* < 0.05).

Roots of plants grown without P exuded more malate and citrate than those in treatments with P added. Neither malate nor citrate showed a significant difference among the treatments with P added (Figure 5A). Plants exuded more citrate than malate only at 0 μM P supply. In other treatments, citrate exudation tended to be less than that of malate. Total carboxylate release declined sharply with increasing leaf P concentration (Figure 5C), but the activity of acid phosphatases showed a gradually-decreasing trend with increasing P supply and leaf P concentration (Figures 5B,D). When focusing on different developmental stages of cluster roots, we found that more citrate than malate was exuded at each stage, but there were no differences between the two carboxylates in root tips. Juvenile-mature and mature cluster roots showed faster exudation rates than root tips and senescent clusters (Figure 6).

### Changes in Rhizosphere pH

**FIGURE 7:**
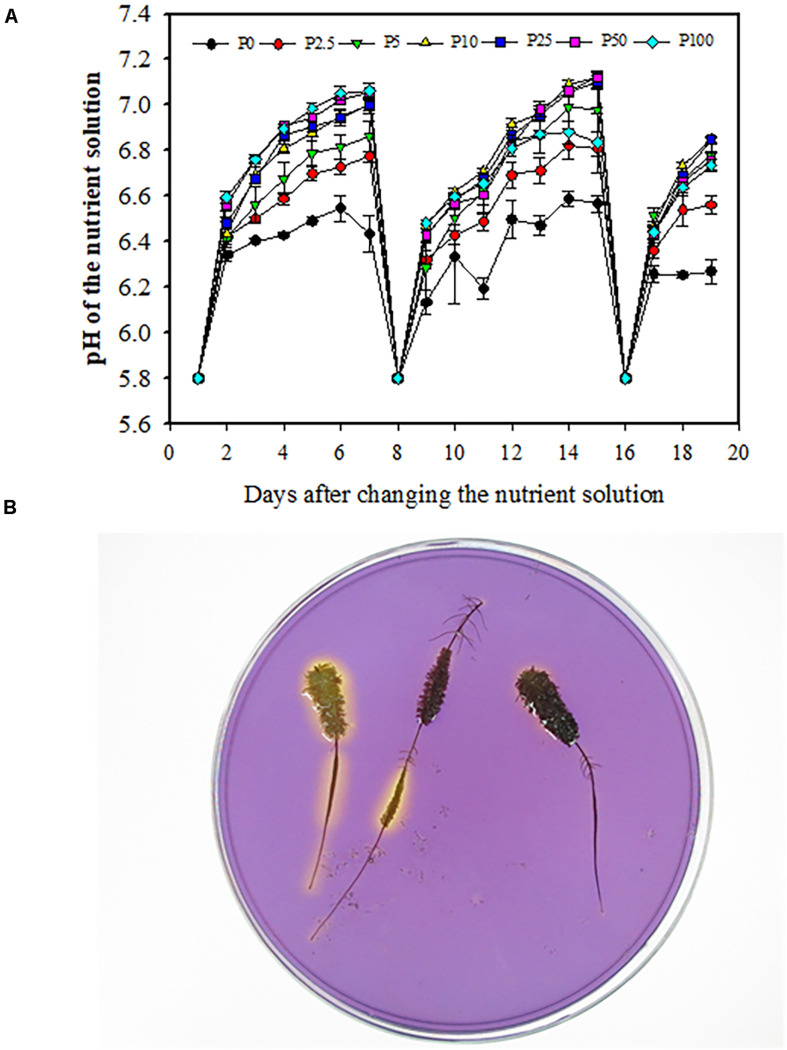
Daily changes of pH in the nutrient solution used to grow *Macadamia integrifolia* under different rates of P supply **(A)**. Using a pH indicator in agar to assess local acidification **(B)**. Solution pH was measured every day using a pH meter. Bromocresol-purple was used as pH indicator. A yellow color indicates acidification. The data represent the means of four replicates ± SE. Different letters indicate significant differences (*P* < 0.05).

Daily tests showed that the pH in the nutrient solution increased every day in all treatments. This trend was most distinct on the first day after changing the nutrient solution (Figure 7A). One day after the nutrient solution was renewed, the pH changed about 0.6 units from 5.8 to about 6.4. In the following 6 days, the change was less, about 0.1 units higher every day. In all treatments, pH in the 0 μM P treatment changed the least, about 0.7 units, during 7 days, while it changed 0.9 units in P2.5; 1.1 units in P5; and 1.3 units in P10, P25, P50, and P100.

Rhizosphere acidification associated with different stages of cluster-root development was shown using the agar method with a pH indicator (Figure 7B). The yellow color surrounding the juvenile and mature cluster roots had a larger range than that around senescent cluster roots, indicating that juvenile and mature cluster roots released more protons from their root surface.

## Discussion

### The Relationship Between External P Supply, Leaf P Concentration, and Plant Growth

This study exhibited clear correlation between external P supply and macadamia biomass and leaf P concentration. Interestingly, we found that biomass of macadamia increased with increasing P supply, with no further increase from 5 to 25 μM P, and a decrease when P supply surpassed 10 μM (Figure 1A). This pattern of macadamia plant growth is supported by previous studies on Proteaceae. For example, the biomass of many Proteaceae increases with increasing P supply in a low P range, but decreases with further P supply due to P toxicity at a concentration that is much lower than used to grow crop plants (Grundon, 1972; Groves and Keraitis, 1976; Lambers et al., 2002; Shane et al., 2004a,b). In a greenhouse pot experiment, *B. ericifolia* shows a positive biomass response to P supply in a range of 30–150 mg P pot^–1^, but its biomass greatly declines at 300 mg P pot^–1^ (Parks et al., 2000). Fresh weight of another Proteaceae, *Banksia menziesii*, is greatest at 1 μM P supply in nutrient solutions and decreases at 10 μM P (de Campos et al., 2013).

In addition to the external P supply, the internal P level, especially leaf P concentration, plays an important role in plant biomass production. The average leaf P concentration of *Banksia* plants growing in a low-P habitat in Australia is only 0.23 mg P g^–1^ DW (Denton et al., 2007), but leaf P concentrations for crop plants are typically 4 mg P g^–1^ DW (Föhse et al., 1988; Lambers et al., 2015a). Leaf P levels of about 0.8–1.0 mg P g^–1^ DW are adequate for macadamia growth (Aitken et al., 1992). Also, leaf P concentrations in a range of 0.7–1.0 mg g^–1^ DW are recommended for macadamia orchards in New South Wales and Queensland, Australia (Huett and Vimpany, 2007). Hue (2009) reported that optimum biomass of macadamia is associated with a P concentration of 1.1 μM P in soil solution and 1.0 mg P g^–1^ DW in leaves. In the present study, shoot biomass of macadamia correlated with leaf P concentration (Figure 3B), and reached the highest value at a critical level of 0.7–0.8 mg g^–1^, which is consistent with previous studies. As previously reported for other Proteaceae (Shane and Lambers, 2005), the leaf P concentration of macadamia also increased with increasing P supply and reached 1.9 mg P g^–1^ DW (Figure 3A). Previous investigations on *H. prostrata* (Shane et al., 2004b), *Banksia attenuata*, and *B. menziesii* (de Campos et al., 2013) showed that excessive accumulation of leaf P caused significant P-toxicity symptoms, which was associated with a limited ability to downregulate their P-uptake capacity. Here, 100 μM P supply was not enough to contribute to significant P-toxicity symptoms in macadamia in this study, except the biomass decreased at 50–100 μM P supply. No leaf symptoms of P tocxicity (brown-gray necrosis on young leaves) were found. Further studies need to be done to confirm whether macadamia can downregulate its P-uptake capacity, as investigated in other Proteaceae.

Our results indicate that macadamia plants are highly sensitive to P supply with a critical leaf P concentration of 0.7–0.8 mg P g^–1^ DW for maximum biomass production.

### Effects of Leaf P Level on Cluster-Root Formation and Exudation

The formation of cluster roots is promoted by P starvation, and suppressed at a high P supply (Lamont, 1972a; Shane et al., 2003a; Lambers et al., 2015a). In this study, cluster-root formation and exudation in macadamia were induced by leaf P starvation, and suppressed by high leaf P levels associated with a high external P supply. A leaf P concentration of 0.7–0.8 P mg g^–1^ DW was a critical value for cluster-root production.

Similar responses have been found in white lupin according to the suppression of the formation of cluster roots after foliar P application (Shane et al., 2003b). Moreover, the formation and functioning of cluster roots in white lupin is regulated by shoot P level in a split-root system. A critical level of shoot P concentration of 2–3 mg P g^–1^ DW was determined which could govern cluster-root formation and citrate exudation (Li et al., 2008). Some species of pasture legume families and *Banksia* from Proteaceae also have the same responses (Denton et al., 2007; Pang et al., 2009; Suriyagoda et al., 2012).

We detected six carboxylates including tartrate, malate, citrate, succinate, fumarate, and *trans*-aconitate. Malate and citrate were the major exudates in every treatment, and succinate was only found at 0 μM P (Supplementary Table S1). Roelofs et al. (2001) found five species of Proteaceae released different carboxylates from their entire root system. Exudation of malate and citrate was strongly stimulated under 0 μM P, as reported for other species bearing cluster roots when growing under low-P conditions (Shane et al., 2004c; Delgado et al., 2014), and citrate was exuded relatively more than other carboxylates (Supplementary Table S1). A possible explanation is that substantial exudation of citrate and malate, especially citrate, has been linked to increased biosynthesis and decreased metabolism of citrate in the tricarboxylic acid cycle (Neumann et al., 1999, 2000; Shane et al., 2016).

The average rate of carboxylate exudation from the whole root system in Proteaceae species occurring in south-western Australia on P-impoverished soils, like *Hakea petiolaris*, *Hakea undulata*, and *Banksia prionotes* is 1.6 nmol g^–1^ FW s^–1^ (5.76 μmol g^–1^ FW h^–1^, Roelofs et al., 2001). Compared with these rates, exudation from the entire system of *Embothrium coccineum* occurring in southern South America in soils that are rich in total P, but with a low available P concentration is nine times faster (Delgado et al., 2014). The difference in the environment has resulted in divergent functioning between *E. coccineum* and Proteaceae from south-western Australia. Total amount of carboxylates released by excised cluster roots of macadamia at 0 μM P was about 11.8 μmol g^–1^ FW h^–1^ (data not shown), and for malate and citrate, the rates were 2.1 and 3.4 μmol g^–1^ FW h^–1^, respectively (Supplementary Table S1). Previous studies reported that the rates of carboxylate release were relatively fast from cluster roots among roots for a range of plant species (Jones, 1998; Roelofs et al., 2001; Delgado et al., 2013). Larger amounts of carboxylate can be released by cluster roots compared with non-cluster roots. Furthermore, a limited number of cluster roots in *Lomatia dentata* were found to have a high exudation rate (Zúñiga-Feest et al., 2020). Carboxylate-exudation rates of excised cluster roots are much faster than those from whole root systems (Delgado et al., 2013). In this experiment, we used excised cluster roots. Thus, the average rate of carboxylate exudation from the whole root system of macadamia could be much slower than 11.8 μmol g^–1^ FW h^–1^, and closer to Proteaceae species in south-western Australia.

Malate and citrate exudation from juvenile-mature and completely mature cluster roots were much faster than those at other developmental stages of cluster roots (Figure 6A). Our results showed no exudation peak in mature cluster roots. The result differs from previous studies on white lupin (Dinkelaker et al., 1989; Watt and Evans, 1999; Skene, 2000) and *H. prostrata* (Shane et al., 2004c), which show an exudative burst for citrate and malate at the mature stage which lasts a few days. We consider two possible explanations. One is that we did not accurately determine the time from initiation of the cluster roots to senescence, thus leading to a possible bias for our classification of the cluster-root developmental stages. Another possible explanation is that the exudative burst of mature cluster roots in macadamia lasted a relatively short time, which we did not capture. Further studies on developmental stages of macadamia cluster roots need to be done.

Our results show that carboxylate release declined sharply when the P supply was increased from 0 to 2.5 μM when the leaf P concentration was about 0.5 mg P g^–1^ DW. The release of carboxylates is likely more sensitive than the initiation of cluster roots for macadamia in response to P supply, as was found for *E. coccineum* (Delgado et al., 2014). Similar to the exudation of carboxylates, the activity of acid phosphatases was higher under 0 μM P. However, the activity of acid phosphatases decreased marginally with increasing leaf P concentration associated with the P supply, indicating that the exudation of carboxylates responded more strongly to P level than the release of acid phosphatases.

### Effects of External P Supply on the Rhizosphere pH

The release of carboxylates is concomitant with rhizosphere acidification, because carboxylate release via an anion channel requires a proton gradient (Tomasi et al., 2009). In the present experiment, acidification occurred in the rhizosphere of clusters of macadamia, especially during the juvenile and mature stages (Figure 7B), as found in white lupin (Watt and Evans, 1999; Shen et al., 2003, 2005) and Proteaceae species, for instance, *E. cantareirae* (de Britto Costa et al., 2016).

We observed an increased pH in nutrient solution compared with the original pH, mainly due to uptake of NO_3_^–^-N. The change in rhizosphere pH depends on nitrogen source (NO_3_^–^ vs. NH_4_^+^) and the buffering capacity of the solution (Marschner and Römheld, 1983). In this study, we used NO_3_^–^ as nitrogen source; uptake of NO_3_^–^ results in the alkalinization of the rhizosphere (Hinsinger et al., 2003; Feng et al., 2020). We also found that P deficiency decreased the pH in the nutrient solution compared with high P supply, during the 6 days after changing the solution, suggesting a stimulating effect on proton release by P-deficiency as reported before (Neumann et al., 1999; Shen et al., 2005). Moreover, cluster roots could acidify rhizosphere, and the treatments of high P supply have little or even no cluster roots. Therefore, weakened acidification occurred in the treatments of high P supply due to the decreased cluster-root number compared with low P supply. The difference in pH change among the P treatments was the consequence of balancing anion and cation uptake as well as nitrate reduction.

## Conclusion

In conclusion, our results suggest that leaf P concentration in macadamia regulates the development of cluster roots and exudation of carboxylates, which is affected by external P supply, and that the plants produce most shoot biomass and show the greatest cluster-root formation and functioning at a critical leaf P concentration of 0.5–0.8 mg g^–1^ DW.

## Data Availability Statement

The raw data supporting the conclusions of this article will be made available by the authors, without undue reservation.

## Author Contributions

XZ, HL, and JS designed the study. XZ and YL performed the experiments and collected the data. XZ analyzed the data. XZ, YL, KJ, HL, and JS interpreted the data and wrote the manuscript. All authors contributed to the article and approved the submitted version.

## Conflict of Interest

The authors declare that the research was conducted in the absence of any commercial or financial relationships that could be construed as a potential conflict of interest.
