# Supplementary material for: Leaf Phosphorus Concentration Regulates the Development of Cluster Roots and Exudation of Carboxylates in Macadamia integrifolia
Source: Front Plant Sci. 2021 Jan 13;11:610591. doi: 10.3389/fpls.2020.610591 (PMC7838356; doi:10.3389/fpls.2020.610591)
Supplement: Supplementary file 1 [file Data_Sheet_1.docx]

Supplementary Material

# Supplementary Data

Article title: **Leaf phosphorus concentration regulates the development of cluster roots and exudation of carboxylates in** ***Macadamia integrifolia***

Authors: Xin Zhao, Yang Lyu, Kemo Jin, Hans Lambers and Jianbo Shen

Fig. S1 Relationship between shoot biomass and shoot phosphorus (P) concentration.

Table S1 Effects of phosphorus (P) supply on root carboxylate exudation of *Macadamia integrifolia*. Plants were grown for six months at a range of P concentrations.

# Supplementary Figures and Tables

Fig. S1 Relationship between shoot biomass and shoot phosphorus (P) concentration.

Table S1 Effects of phosphorus (P) supply on root carboxylate exudation of *Macadamia integrifolia*. Plants were grown for six months at a range of P concentrations.

| P supply | tartrate | malate | citrate | fumarate | T-aconitate | succinate |
| --- | --- | --- | --- | --- | --- | --- |
| (μM) | (μmol g^-1^ FW h^-1^) | | | | | |
| 0 | 0.63±0.16 | 2.12±0.63 | 3.39±0.84 | 0.04±0.01 | 0.04±0.01 | 5.57±2.89 |
| 2.5 | 0.56±0.33 | 0.44±0.09 | 0.40±0.11 | 0.06±0.03 | 0.002±0.001 | - |
| 5 | 0.11±0.03 | 0.44±0.22 | 0.10±0.04 | 0.06±0.04 | 0.019±0.005 | - |
| 10 | 0.20±0.02 | 0.64±0.26 | 0.46±0.04 | 0.01±0.00 | 0.003±0.001 | - |
| 25 | 1.37±0.85 | 1.11±0.14 | 0.20±0.09 | 0.02±0.00 | 0.006±0.001 | - |
| 50 | 0.33±0.13 | 0.36±0.14 | 0.12±0.05 | 0.01±0.00 | 0.004±0.001 | - |
| 100 | 0.05±0.00 | 0.33±0.11 | 0.07±0.00 | 0.00±0.00 | 0.003±0.00 | - |
